# Epidemiological survey of suicide cases registered in covered hospitals of Isfahan Health Center in 1395 

**Published:** 2019-08

**Authors:** Hamid Turkzadeh, Alireza Dadaei shahrekordi, Corose Aminian, Fatemeh Asgari

**Affiliations:** ^*a*^Isfahan Health Center No. 1 - Center Manager - Isfahan University of Medical Sciences, Isfahan, Iran.; ^*b*^Unit for Combating Illness- Isfahan Health Center No. 1 - Isfahan University of Medical Sciences, Isfahan, Iran.

## Abstract

**Background:**

More than 30,000 people die every year in the United States, and the number of suicides in excess of 650,000 people is now among eight major deaths in the United States. The rate of suicide among different nations varies from 25 to 10,000 in Scandinavia, Switzerland, Germany, Austria the countries of Eastern Europe and Japan to less than 10 in 10,000 people in Spain, Italy, Ireland, Egypt, and the Netherlands. Suicide in Iran by the forensic organization in 1380 suggests that 5.7 suicides per 100,000 men and 3.1 suicide per 100,000 women occur in the country. Statistics show that suicide rate in men in European and North American countries and South-central Asian countries is lower and in Western Asia is higher than other countries. Furthermore, statistics show that Iran and Georgia have the highest rate of suicide in western Asia.

**Methods:**

This was a retrospective descriptive study. The aim of this study was to investigate the epidemiologic pattern of suicide cases referred to hospitals affiliated with Isfahan Health Center No.1 in 1395. The statistics were collected by examining the accident and accident forms and then analyzed using SPSS software package.

**Results:**

Of 21239 incidents, 3371 (15.8%) were related to suicide and the second highest accident rate occurred in urban areas, 50% were males and 50% were women. 98% of suicides occurred in urban areas and 2% in rural areas. In terms of the place of suicide, most cases occurred with 99% at home. The highest rate of suicide was found in the age groups of 20-29 years (39.5%), 30-39 (28.6%), 10-19 years (17.6%), 40-49 years (10.3%) and 50-59 years (2.3%). More than 99% of suicides was treated and only 12 people reported death.

**Conclusions:**

The results of this study indicate that suicide among accidents has a high percentage which requires a special look as one of the components of social health. However, the existence of suicide at a young age and aged generational and active community of alarm Serious for mental health in the community. Since most suicides have occurred in urban areas and there are several factors such as stress, economic problems, lifestyles and other cultural and economic factors involved in it, more research is needed to accurately identify the causes and factors of suicide and find ways to prevent it. This is underlined by this article.

**Keywords:**

Suicide, Active age group, Prevention

